# Mucous Membrane of the Lungs

**Published:** 1820-12-01

**Authors:** 


### Medico-Chirurgical Review,

AND

### JOURNAL OF MEDICAL SCIENCE.

(Analytical Series.)

Nec tibi quid liceat sed quid fecisse decebit
Occurrat mentemque domat respectus honesti. CLAUD.

VOL. I.] DECEMBER 1, 1820. [No. 3.

### MUCOUS MEMBRANE OF THE LUNGS.

### I.

1. *A Treatise on Inflammation of the Mucous Membrane of the Lungs; to which is prefixed an Experimental Inquiry respecting the Contractile Power of the Blood Vessels, and the Nature of Inflammation.* By CHARLES HASTINGS, M.D. Physician to the Worcester Infirmary, &c. One vol. 8vo, pp. 420. London, 1820.

2. *Histoire des Phlegmasies, ou Inflammations Chroniques, fondées sur de nouvelles Observations de Clinique, et d'Anatomie Pathologique.* Par F. J. V. BROUSSAIS, Docteur en Medicine, &c. &c. Two vols. 8vo, 1232 pages. Second Edition. Paris, 1816.

3. *Observations on the Inflammations of the Mucous Membranes of the Organs of Respiration.* By THOMAS ALCOCK, Esq. Surgeon. (Medical Intelligencer, No. 7 & 8.)

4. *Dictionnaire des Sciences Medicales.* Tome 32, Article "*Membrane.*" Par L. R. VILLERME, M.D.

5. *An Essay towards a Pathology of Eruptive Fevers. Delivered as a Gulstonian Lecture, before the Royal College of Physicians, London, in the year* 1820. By RICHARD HARRISON, M.D. F. L. S. Fellow of the Royal College of Physicians, &c. &c &c.

[London Med. and Phys. Journal, July 1820.]

THE current of medical investigation and curiosity has lately set, with considerable force, towards the pathology of the Mucous Membranes—more especially those which line the aerial passages. This current of attention may appear to be too strong, and too exclusively directed towards a single point; but we believe that medical science is not likely to be retarded, in the end, by such accidents. A certain degree of enthusiasm seems necessary to ensure success in all pursuits: and it would be bad policy to repress it in a profession like ours, where there exist already so many circumstances to chill the zeal, damp the ardour, and exhaust the patience of those who devote their time and talent to the investigation of diseases.

It is now thirty years since Theophilus Bordeu, of Montpellier, published a volume on the "*Tissu Muqueux, ou l'[illegible]ane Cellulaire, et sur quelques Maladies de la Poitrine;*" b[illegible]e confess that an attentive perusal of this work has furnished us with little knowledge on the subject. M. Pinel appears to have been the first who drew order out of chaos respecting the different tissues of the body; and Bichat, seizing the idea, carried his investigations (as far as anatomy and physiology are concerned) to the utmost extent; for very little has been added to the researches of this powerful genius. A slight anatomico-physiological sketch of the tissue which forms the subject of this article is indispensably necessary towards a pathological delineation.

The mucous membrane which lines the air passages of the lungs, and the whole alimentary canal, from the mouth to the anus, is composed of three tissues or structures—the chorion, papillæ, and epidermis. This membrane forms a kind of internal skin, and is strikingly analogous to the cutaneous surface, in its organization, functions, and vital properties. The chorion of the mucous membrane, which gives form and substance to this tissue, is of a soft and spongy texture, which Bichat conceived to be very much modified by its own secretions, as well as those foreign substances which constantly come in contact with it. It is curious that all the mucous surfaces, especially those of the stomach, have the property of coagulating milk, as is commonly seen in the process of making rennet with the paunches of animals. The papillæ or villi of the mucous membrane are so numerous and minute, that they generally appear as a polished surface, yet their existence is unquestionably proved. They are of a pyramidal shape, and very various in size, in different parts of the mucous tissue. The free or internal surface of this membrane is covered with an epidermis, analogous to that of the skin, but very fine, insensible, and apparently for the purpose of defending the chorion and papillæ from the rude action of passing substances. Throughout the whole extent of the mucous membrane, and embedded in the chorion, are scattered innumerable glands, which secrete and pour out, by imperceptible orifices, a mucilaginous fluid, which lubricates their surface. These glands are very vascular, and doubtless are proportionally supplied with nerves. Whenever the mucous membrane is irritated the function of secretion is increased. The greatest degree of sensibility resides at the extremities of the mucous membranes, and any stimulus applied there excites the action of the mucous glands throughout the whole extent of tissue. This may account for many phenomena which we daily witness: thus the introduction of a suppository, or even a bougie into the rectum, will often excite the action of the whole intestinal tube. Bichat, instead of stimulating the cutaneous surface of a hemiplegic patient, has, more than once, pursued the following plan. He introduced a sound into the urethra, and a bougie into each nostril, while a surgeon irritated, at intervals, the uvula. The patients appeared to be much more roused by these means than by blisters to the skin. "Would it not be better (says Bichat) to produce an artificial catarrh in the mucous membrane of the nostril corresponding with an inflamed eye, than apply a vesicatory externally?"

As the mucous membranes are constantly exposed to the contact of substances extraneous to those of the animal, we may perhaps consider one office of them to be that of interposing a limit or barrier between our organs and those heterogeneous bodies—in short, officiating as a skin to the internal viscera. These mucous surfaces, when they happen to protrude without the body, as for instance, in inversio uteri, appear to take on the nature of common skin, in a great degree. In the impunity with which they may be exposed to the air, they exhibit a striking contrast with the serous membranes of the body. Into the structure of the mucous membranes a vast number of blood-vessels, nerves, exhalents, and absorbents are crowded, and no tissue in the human frame is endued with a higher degree of excitability, or irritability. Each mucous membrane, however, is specifically excited by a specific substance—as that of the bladder by urine, that of the lungs by air, &c. But when their organic sensibility is disturbed by disease, even their proper fluids will occasion pain, as we every day witness.

When we contemplate the great extent of the mucous surfaces, their importance, even as emunctories, which separate their fluids from the blood, rises in our consideration. Any excess or deficiency in their functions must, and evidently does, derange the general health in a very powerful manner. But their extensive chain of sympathies with almost every structure in the body, especially the skin, deserves the utmost attention of the physician; for it is principally in this way that we can trace the immense range of their influence on distant functions and structures of the human frame. In a future number we shall investigate exclusively the mucous membrane of the digestive apparatus, and then will be the proper time for taking up the subject of morbid sympathies. At present we confine our pathological researches and observations to the membrane lining the air passages.

Some physiologists believe that the mucous membrane of the lungs plays an important part in the change which is produced in the blood while passing through the pulmonary capillaries. The exhalation from the mucous tissue of the lungs is sufficiently obvious; but whether the fluid exhaled be merely aqueous, or whether any of the mucous fluid can be converted into vapour at the temperature of the human body, is doubtful. Indeed we are far from being assured that the fluids which lubricate the pulmonary surfaces in a state of health, are of that viscid nature which we observe them to be under other circumstances. It is, on the contrary, exceedingly probable that this viscidity prevents, in some measure, that change in the blood, usually effected by respiration, and, in many cases, goes far by its tenacity and accumulation to prevent that change entirely, and thus destroy life. In perfect health there appears to be no expectoration—the fluid secreted being completely exhaled or absorbed.

It is remarked by Bichat, that where the mucous membranes take their origins from the skin, and where their animal sensibility is most exquisite, they are there supplied with nerves from the cerebrum; as, for instance, the pituitary membrane, the conjunctiva, &c. while the deep-seated portions of the mucous membranes are supplied chiefly by the ganglia, as the intestines, excretory ducts, &c. Although, in health, the air and aliments produce no sensation in the mucous tissues to which they are applied, excepting at the origins of these tissues, as above-observed, yet, when they are in a state of inflammation, the same substances produce much uneasiness; and in this state a great quantity of first watery and then viscid mucus, or even pus, is poured out, as we see in pulmonary and vesical catarrh, dysentery, &c. There can be no doubt that this increased secretion is the means which nature employs for the removal of the irritation or inflammation, in the same way that a watery fluid is poured out from the serous membranes, to relieve their vascular turgescence. Yet an excess in either of these processes is attended with great danger. "No sooner does the secretion into the air passages exceed that which can be removed by expectoration, than accumulation begins, and a part of the cells of the lungs, which should receive air, becomes filled with secreted fluid. This increasing must necessarily prevent the due performance of the function of respiration—that function without which life cannot exist. The blood no longer is changed from the dark or venous to the vermillion hue, and the colour of the body partakes of the leaden or livid shade;—if the accumulation proceed, respiration becomes more and more obstructed, the phlegm may be heard rattling in the air passages—and the patient sinks exhausted and suffocated."*

It is known to our readers that, in this country, Dr. Harrison, Dr. Hastings, Mr. Alcock, and a few others, have recently drawn the attention of the profession to the pathological affinity of certain eruptive and other diseases with inflammation of the mucous membranes. This affinity has, for some years, been observed, and fully acknowledged on the continent.

"It is," says Villermé, "to irritation of the mucous membranes, particularly those of the digestive organs, that we are to attribute a great many of the cutaneous phlegmasiæ, hitherto regarded as idiopathic affections. The miliary eruption appears to be always symptomatic. We invariably find, in individual cases, and in the histories of epidemic miliary fevers, that all the symptoms of irritation in the mucous membranes preceded the cutaneous eruption, and coincided with it. On dissection too, the physician has always found unequivocal traces of irritation and violent inflammation of some mucous membrane, falsely regarded as the *effect*, but evidently the *cause* of the miliary eruption. It is equally certain that in measles the mucous membranes are primarily affected, and that, from their tissue the irritation is reflected on the cutaneous surface:—in short, that the measles is only an epiphenomenon or appendix of the internal irritation. La Rougiole n'est qu'un appendice de l'irritation interne." *Dict. des Sciences Med. Tom.* 32, *p.* 218.

In our review of Dr. Clark's work, April 1820, p. 587, we noticed the investigations of our continental brethren relative to the pathology of small pox. All those who died of this disease at the "Maison des Enfans" (and the mortality was great) were examined, and the appearances were inflammation and ulceration of the internal coat of the intestines; with pustular eruptions there, and on the surface of the peritoneum. In three cases a croupy false membrane was found lining the whole alimentary canal, from the œsophagus to the rectum. Mr. Alcock, whose dissections shewed the mu-

* Alcock.

cous membrane of the lungs more frequently affected, in variola, than that of the digestive organs, suspects that the French physicians did not examine the air passages. We have reason to know, however, that they left no important part of the body unexplored. But the difference between the climate of England and France, together with the heating regimen which, for a time, was employed in the MAISON DES ENFANS, will easily account for the mucous membrane of the lungs being the principal focus of disease in this country, while that of the digestive apparatus bore the onus of irritation in Paris.

Mr. Alcock, whose opportunities for prosecuting investigations of this kind are great, and whose originality of mind qualifies him for the task, observes that:

"In measles we trace the first appearances in the watery and suffused eyes—the discharge from the nostrils—the sneezing and other catarrhal symptoms, which shew the inflammation of the mucous surfaces lining these parts: the inflammation extends inwards, and the throat becomes affected; as is evident, on inspection, and as might be inferred from the greater or less difficulty in swallowing. The sneezing is generally a mere precursory symptom, whilst the cough, which is at first generally slight, is often increased to a severe degree, shewing the transition of the diseased action from one part of the mucous surface to another. In some cases these symptoms are much relieved on the appearance of the eruption, but in most of the severe forms of this disease it is otherwise, and the vulgar opinion that the greatest danger is to be apprehended as late as the decline of the eruption, or as is usually expressed *at the turn*, seems not without foundation. If the disease have proceeded thus far without appropriate treatment—if there be much dyspnœa, and the cough be frequent and severe—if the breathing, besides being oppressed, be much increased in frequency beyond the natural standard, the result is indeed much to be feared." *Med. Intell. Vol.* I. *p.* 197.

Mr. Alcock thinks that, during the progress of measles, we may as certainly infer the state of the lining membrane of the lungs, by a careful inspection of the fauces and adjacent parts, as we may infer the state of the stomach from that of the tongue.

"Examinations of morbid appearances, after death, in cases of measles, have shewn the lining membrane of the air passages in a highly vascular and inflamed state; the air passages themselves filled in so large a proportion with mucus, as to prevent the oxydation of the blood, and consequently to destroy life. In cases where the disease has been more protracted, the air passages have not been filled with mucus, but chiefly with pus, sometimes mixed with air and mucus, extending throughout the most minute ramifications of the bronchia, and rendering a great part of the lungs much more dense than in the natural state." *Med. Intell. p.* 197.

The parenchymatous structure of the lungs did not appear to suffer much, in Mr. Alcock's experience; but he has known more than one instance where ulceration of the larynx had taken place. During the period of eruption he not unfrequently found the larynx and pharynx principally affected, while, in the more advanced stages, the trachea, and still more frequently, the bronchia became the chief seat of disease. Mr. A. observes, that the greater number of these affections are acute, and require speedy and efficient means of relief; whereas inflammations of the same tissues, unconnected with these eruptive complaints, often continue for a considerable time without danger. Mr. Alcock, throwing all idea of the specific nature of measles (and indeed of other eruptive diseases) out of the question, and viewing the malady simply as inflammation of the mucous membrane of the lungs, founds his treatment, first, on the controlling of the general excitement; and secondly, on subduing the local inflammation. The means of fulfilling these indications need not be stated to the readers of this journal.

In respect to small pox, as was observed above, Mr. Alcock found the aerial much more frequently affected than the alimentary passages; and he states it as a general fact "that the danger of small-pox has always been in the ratio of the laryngeal, tracheal, and bronchial inflammation, and that this inflammation has usually borne a strict relation to the apthous eruptions which are to be found in the throat."* A reference therefore to the state of the throat, in the early days of the eruption, has proved an almost unerring guide to Mr. Alcock, in his future practice. "In nearly all cases of confluent smallpox, when seen thus early, a decidedly depletory and antiphlogistic system of treatment has conquered the disease, and saved the person's life."

"So certainly has this treatment answered the intended purpose, that in many cases the disease has been cut short, the eruptions have

* Very many years ago Reil came to nearly the same conclusions. "Sur quinze ouvertures de sujets qui avaient succombé a variole, Reil a trouvé onze cas où les bronches étaient attaquées de phlegmasie, soit seules, soit conjointement avec le larynx, la trachée ou le poumon; et tantot elles contenaient un mucus de couleur pourpre foncée, ou du sang aqueux, ou une matière epaisse, fètide et noirâtre; tantot leur glandes étaient tres gonflées quelquefois ces tuyaux ne presentaient qu'une legere phlogose avec des taches rouges; d'autres fois une fausse membrane plus ou moins epaisse, blanchatre et tenace, recouvrait la tunique interne du tube aerien très inflammée." *Dict. des Sciences Med. Vol.* II. *p.* 130. We cannot lay claim to any great novelty in our mucological doctrines of the present day, after the passages quoted in this article are fairly considered.

run through their course more rapidly than under ordinary circumstances, the secondary fever, as it has been called, has not occurred, and the person's countenance has been left free from pits, or the marks have been comparatively slight." *Med. Intell. p.* 201.

Dr. Hastings, of Worcester, whose work will presently come into particular consideration, relates two cases of fatal variola, where the mucous membrane of the lungs was inflamed or ulcerated. In the first case, (p. 238) a child, three years of age, after exposure to the contagion of small-pox, became feverish on the 14th of November. On the 17th a red pimply eruption appeared on several parts of the body. On the 19th there was some difficulty of breathing, which, next day, was increased, and he died the same evening. On dissection, the mucous membrane of the pharynx was found inflamed; while the trachea, bronchia, and air cells were quite filled with mucous and bloody serum. The membrane lining the trachea and bronchia was greatly inflamed; but the substance and pleural covering of the lungs were sound.

The brother of the foregoing, six years of age, was seized with variola, which proved fatal on the 6th day, and without any very evident pectoral symptoms. He complained, however, of soreness of the throat, and some fits of dyspnœa. On dissection, the pharynx and upper part of the glottis were found sphacelated. A quantity of bloody matter escaped from the trachea when incised. The mucous lining of the trachea and air-cells was much inflamed; and about the middle of the trachea there were several small ulcers. The bronchia and air-cells were completely full of a bloody fluid.

"It is common," says Dr. Hastings, "if death happen at an early stage, before the vescicles are formed, to find the trachea full of fluid, and the mucous membrane of the lungs exceedingly red from the dilatation of its capillary vessels. The glottis too, and the upper part of the trachea, are sometimes highly vascular, and the inflammatory action often extends to the pharynx; but neither the pleura nor the substance of the lungs appears inflamed. If the patient die at the later period of the disease, when the body is covered with pustules, ulcers are occasionally met with in the mucous membrane, corresponding in size with the pustules that are formed in the skin. The glottis and pharynx are still more affected than when the patient dies earlier, sphacelus of those parts occasionally occurring. The structure of the lungs is often entirely free from disease, and there are no adhesions. The lungs, however, do not collapse when the thorax is opened, and when cut into, a frothy matter escapes from them. The blood vessels of the lungs are always much loaded." 185.

Mr. Cross and Mr. Hull of Norwich have also stated some dissections after small pox, where an eruption of a variolous appearance was found on the inner surface of the stomach, as the reader will see in our last number, page 305.

In respect to hooping cough, little doubt is now entertained of its being a specific inflammation, more or less acute, of the pulmonary mucous membranes—especially that portion lining the larynx.

"I have repeatedly ascertained," says Mr. Alcock, "by dissections of patients who have died of hooping cough, that the larynx invariably exhibited signs of inflammation often to so great an extent as by its swelling to close mechanically the glottis;—often the exudation of coagulated lymph near the larynx; the mucous or lining membrane of the trachea and bronchia much increased in vascularity, and the cavities of the latter filled with fluid more or less mixed with air,—the fluid varying from that of thin mucus to perfectly formed pus.

"With these facts as a basis, is it unreasonable to suppose that the inflammation of the mucous membrane lining the various parts of the organs of respiration, should produce the tenacious mucus, which is from time to time expectorated? that the accumulation of this matter impedes respiration, and acts as an extrananeous body upon the larynx; that the cough is a mere natural effort to expel the offending matter, and its violence is in direct ratio with the tenacity of the phlegm secreted;—as we often find that spontaneous vomiting frequently terminates the paroxysm, by bringing away the secretion adhering about the top of the larynx, and which the cough had not been sufficient to dislodge? This irritating cause being removed, there is soon a cessation of all the urgent symptoms until its accumulation, or other accidental cause, produces the recurrence of the paroxysm. If the disease be not subdued, either by natural means or by appropriate remedial treatment, the excessive secretion continues, accumulation takes place in the bronchia and in the air cells of the lungs, and consequently the blood is prevented from that intimate contact with the air respired, which is so essential to life, that the continuance of this state terminates in suffocation."—*Med. Intell. p.* 202.

The treatment, Mr. Alcock observes, must not be confined to the mere alleviation of symptoms, but must, to be successful, strike at the cause. When the inflammatory affection appears to call for measures beyond those of regimen, temperature, &c. "depletion, active depletion must be used; and frequently the combination of blood-letting, both general and local, will afford more relief than either of them separately." These means will be greatly assisted by such remedies as tend to equallize the circulation; the distribution of blood being generally in excess in the internal organs, and deficient on the surface. The occasional exhibition of emetics is therefore useful, "and still more particularly such medicines as produce a copious mucous secretion from any large surface of the mucous membranes, as those of the stomach and intestines."

Dr. Richard Harrison, in a late Gulstonian Lecture before the college of physicians, subsequently published in the Medical and Physical Journal, has drawn the attention of his brethren to the connexion between eruptive fevers, and inflammation of the mucous membranes. While ingeniously supporting this doctrine, however, he has gone a step beyond Broussais himself, by supposing "that it is irritation of *one organ solely* that will produce this effect, (fever) and that organ is the mucous membrane." This is denying the power of any other inflamed organ; as, for instance, the brain, to produce symptomatic fever, until the disorder has reached the mucous membranes, when "derangement of certain functions of the body," hitherto denominated fever, becomes immediately developed. It is somewhat mortifying to human, and particularly to medical speculations, that two learned and ingenious physicians, in the present advanced æra of pathology, should, in the British capital, promulgate two theories respecting fever—one maintaining that it depended *always* on inflammation of the brain—the other, that it *never had* such a source. Our own observations do not lead us to coincide entirely with either Dr. Clutterbuck or Dr. Harrison. The etiology of fever is too various to admit an unvarying pathology of the disease. Truth would appear to lie between these exclusive extremes; for, granting that fever was always dependent on topical inflammation, that topical inflammation will not be found exclusively confined to a single organ or structure in the body.* Nevertheless the public are greatly indebted to those gentlemen who have endeavoured to assign fever a local habitation, since the doctrine points to by far the best practice in the majority of cases. If we could bring ourselves to the doctrine of an *exclusive seat* of fever, we should certainly be inclined to consider the mucous membranes as that seat, for it appears far more generally affected than any other tissue in the body; but we are not converts to the doctrine, notwithstanding the ingenuity and acuteness of its advocates.

As far as respects the eruptive fevers, however, we are strongly disposed to agree with Dr. Harrison and those other practitioners already alluded to, that the primary exciting cause, be it a specific contagious miasm, or certain unknown peculiarities of atmosphere, is first applied to the mucous

---

* Med. and Phys. Journal, July 1820, p. 17.

membranes of the lungs and primæ viæ, and that in the said tissues we have the first indications of disordered function, during life, and very unequivocal vestiges of inflammation, on dissection.

"In fact," says Dr. Hastings, "the membrane (in measels) is sometimes affected in patches nearly approaching to the figure of semicircles, in the same manner as the skin is in that disease." 193.

Under such circumstances, and knowing as we do, how commonly a disordered state of the internal secretions will affect the skin, we may fairly conclude that the febrific miasms which occasion the eruptive fevers take the route above mentioned in their action on the human frame. But although we conceive that inflammation is the danger to be guarded against, and the point to be held in view, while treating the eruptive fevers; yet we cannot bring ourselves to agree entirely with those who would identify *this* inflammation, as far as treatment is concerned, with inflammation occurring under common circumstances, and independent of any eruptive disease. It is quite evident that in the exanthemata Nature expels the poison, or, at all events, produces a metastasis to the skin—and this she does not appear to attempt in *common* inflammations. Some little deference then, we conceive, is to be paid to Nature in this specific operation, and her work should not be rashly interfered with, until she appears to be going wrong. We find that, under common and favourable circumstances, Nature conquers the internal inflammation in her own way; we think therefore that it is only where the interior functions labour, and the internal structures are threatened with disorganizing inflammation, that we are to act as boldly as in the common phlegmasiæ of the internal organs. In the latter case, we are convinced that nothing but pure antiphlogistic measures can be effectual, and those put in force with vigour, not timidity.

Having thus glanced at the pathology of the mucous membranes in the exanthemata, we shall proceed to that of the lungs, as more commonly and obviously affected under other circumstances.

The respiratory organ is more exposed to external impressions, especially of climate, than any other viscus in the body; and its lining or mucous membrane, which is the most sensible structure of this complicated apparatus, is evidently the first surface on which these impressions can be made. It is to be borne in mind that whatever raises the sensibility or irritability of the mucous membrane of the lungs (or indeed of any other tissue) beyond the natural standard, augments proportionally the vascular fulness of the part; and, on the other hand, whatever determines an overplus of blood to this membrane, elevates its sensibility beyond the healthy boundary. Now in variable climates particularly these oscillations in the circulation and excitement of the mucous membrane are almost hourly produced. All irritating substances diffused in the atmosphere and drawn into the lungs, excite the nerves of their lining membrane; and this excitement draws an afflux of blood to the part—while every considerable alternation of temperature in the air, impels from the surface of the body an increased quantum of blood to the interior organs generally, but especially to the mucous membrane of the lungs, accompanied, or quickly followed, by a corresponding afflux of sensibility. This is no imaginary doctrine. Common observation pointed it out among the snow-covered mountains of Styria, when the legions of Napoleon were overrunning the Continent.

"Toutes les fois," says Broussais, "que la température de l'air ou de l'eau vient á diminuer autour de l'homme, qui, d'ordinaire, baigne dans ces fluides, la peau se refroidit, ses évacuations cutanées diminuent, le sang est en moindre quantité dans l'organe cutané, dans le tissu cellulaire et dans les membres; il abonde dans la muqueuse pulmonaire, ce qui est démontré par un sentiment de plénitude qu'on éprouve en inspirant, dans la poitrine; l'exhalation et la sécrétion muqueuse y sont augmentées. Si, après la cessation de la cause, l'équilibre n'est pas rétabli, l'homme a une irritation morbifique dans la membrane des bronches."—*Tom.* I. *p.* 140.

In the various avocations of life there are numerous other auxiliary causes in the production of these irregularities of the circulation and excitement in the pulmonary apparatus. Inordinate exertions of the body or of the voice continually strain the vessels of the lungs; and, where any predisposition exists, excite a tendency to inflammation or congestion. And this leads to inquire what are the symptoms of this inflammation? Are we to consider every excitement of the mucous membrane of the lungs, as evinced by an increased secretion from its glands, as inflammation? Or, would it not be more correct pathology to consider *irritation* as the first link or grade, which may or may not rise into actual inflammation of the mucous structure? Volatile alkali or snuff will excite a considerable secretion from the mucous membrane of the nares, and upper portions of the larynx and fauces, with sneezing or coughing; but are we justified in calling this inflammation? Fruit and various other ingesta will often so excite the mucous membrane of the bowels as to occasion a smart diarrhœa, but is this inflammation? We think the following sentence of Broussais is worth attending to:—

"If this irritation exhibits no other indication of its existence than a disordered mucous secretion (un vice de la secretion muqueuse)

it is called *catarrh;* but if it manifests itself by a violent disturbance of the circulation, joined to an alteration in the mucous secretion, we call it pulmonic inflammation."—*Tom.* I. *p.* 140.

This is probably all the distinction which we at present possess, between irritated function and inflamed structure. We are carefully to bear in mind, however, that inflammation almost invariably commences with irritation of function; and that irritation of function readily passes into inflammation or other disorder of structure.

We now come to the subject of Bronchitis, and here we take up the work of Dr. Charles Hastings, a gentleman whose zeal and talents will, we venture to prognosticate, do honour to the present æra of pathological investigation, and contribute greatly to the progress of the healing art.

Dr. Hastings has presented to the reader a concise yet clear exposition of what has been written on the subject of bronchial inflammation, previously to his own time, on nearly the same plan as Dr. Cooke has adopted on the subject of nervous diseases—a mode of proceeding, in both cases, which we highly recommend to the attention of our brethren, as deserving their warmest patronage and support.

Dr. Hastings very properly divides bronchitis into two kinds, the acute and chronic, together with the varieties of each kind which present themselves in practice.

*Symptomatology of Acute Bronchitis.* § 1. Dr. H. considers catarrh as the lowest grade, or mildest form under which bronchial inflammation is observed. The symptoms of this affection are well known, as well as its salutary termination in a mild mucous expectoration, which relieves the turgescence of the vessels, and permits the membrane to regain its pristine integrity of function. It is not very uncommon, however, for highly inflammatory symptoms to supervene on catarrh, occasionally, or the disease to degenerate into an obstinate chronic affection, in delicate constitutions, and under mismanagement or neglect. The cough then becomes severe, the expectoration copious and puruloid if not purulent, the dyspnœa considerable, the pulse quick, while the patient emaciates and exhibits most of the phenomena of phthisis, which, if not checked, prove fatal.

On dissection, in such cases, according to Dr. Hastings, tubercles are generally found in the lungs, and the mucous membrane much diseased, viz. ulcerated, and the bronchia and air cells filled with purulent matter.

§ II. The second variety happens generally during sudden atmospherical vicissitudes, in old people, and phlegmatic habits. The apparent mildness of the attack is fallacious; the febrile symptoms are not near so strongly marked as in pneumonia; there is little or no fixed pain in the chest, but only a sense of uneasiness and tightness; there is oppression at the præcordia, with anxiety in the countenance, lassitude over the body, quick and laborious respiration, wheezing, and, as the disease advances, a noisy breathing, from the accumulation of the secretions in the air cells and bronchia. Hoarseness is common; and the patient cannot breathe deeply without exciting cough, or increasing pain, if any existed. In the early period of the disease, the dyspnœa is not much aggravated by the recumbent posture; but as it advances, the respiration is freer in the erect position of the body. Dr. Badham remarks, that in some cases of asthenic bronchitis, there are periodical exacerbations of the dyspnœa, resembling transitory fits of asthma, with sudden constriction across the thorax, and momentary loss of voice. At the very beginning, there is often a dryness of the mucous membrane, the secretions being diminished: but cough is always present, and some expectoration usually attends the progress of the complaint. When a copious expectoration comes on of thick, viscid, opaque mucus, the violence of the cough is generally diminished. Vomiting not unfrequently is excited by the cough in bronchitis, and the discharge of dense white mucus, sometimes moulded into ramifications, usually relieves the bronchia for a time. After violent paroxysms of coughing, the patient is often left breathless, with a sensation of tightness across the chest. Great pain across the forehead generally accompanies the cough—often vertigo and drowsiness. The tongue and stomach are deranged, and there is thirst. At the very beginning the circulation is not much disturbed; but in the progress of the disease, the fulness and hardness of the pulse are perceptible. The temperature of the body is not, in general, much increased, though the cheeks are often flushed, with evening paroxysms of heat and restlessness. The surface is dry, unless when acted on by diaphoretics, and blood drawn usually shews the buffy coat. The duration of this species of bronchitis is very uncertain.

"In some cases," says Dr. Hastings, "it terminates in a few days, whilst in others it runs on to a much longer period. In the more violent examples, when the remedies employed do not check the progress of the symptoms, the pulse towards the seventh or eighth day becomes very quick and much weaker; occasional perspirations break out, the nails and lips assume a slightly livid hue, and the countenance is distressed, anxious, and pallid, with somewhat of a purple tinge. In fact, every symptom bespeaks obstruction in the air passages. Soon after the extremities grow cold, and the patient dies from suffocation." 163.

In less dangerous cases the more distressing symptoms begin to subside in six or seven days, with a copious, bland expectoration, which generally continues for some time, and prevents the patient from recovering his health for several weeks. In this state unfavourable weather may fix a chronic and obstinate affection of the mucous membrane, which occasionally destroys the patient with all the symptoms of phthisis, but without disorganization of the parenchymatous structure of the lungs, as will be shewn hereafter. Hydrothorax is not a very unfrequent termination of this complaint when long protracted.

§ III. Hitherto our judicious author has been considering the disease as of a comparatively mild, catarrhal nature, or in old and phlegmatic subjects, where the pyrexia does not run high, and the disease is not very rapid in its course. But when the subject is strong and plethoric, the symptoms assume a tone of great severity. While the pulse, tongue, and surface indicate violent reaction, the countenance is often pallid. Even here there is rarely any fixed pain, but only a distressing tightness in the chest. The breathing is laborious, and only mitigated by the erect posture. The dyspnœa is far greater than the cough. There is some expectoration; a failure of which is a most unfavourable symptom. This stage of excitement, if not relieved, almost always terminates in a corresponding collapse of the system; when we have orthopnœa, purple lips, sunken and quick pulse, diminished temperature of surface, cold perspiration. The expectoration now becomes scanty, or ceases altogether, and the patient dies suffocated by the accumulation of secretions in the air cells and passages, often as early as the 5th or 6th day.

Vigorous measures at the beginning occasionally check the dangerous symptoms; but the great debility necessarily induced renders recovery slow, and a tedious chronic disease too often ensues, characterized by frequent and violent cough, with a very copious expectoration of puruloid matter, and, in fact, all the symptoms of pulmonary phthisis, sometimes terminating favourably, when aided by climate or season; often carrying the patient off in a state of marasmus and hectic fever.

§ IV. Young children are subject to acute bronchial attacks more speedily fatal, though not apparently so severe or dangerous as the last described variety. It is most common in the spring, commencing with, and retaining the catarrhal character. The breathing is somewhat quickened, with wheezing, but not much dyspnœa, nor uneasiness from supination. The cough is slight, and there is but little expectoration, except when vomiting is induced. The appetite is generally preserved, and the fever is moderate, but the pallor of countenance is remarkable from the beginning, with frequent, and sometimes hard pulse. In the progress of the disease the breathing becomes very variable—sometimes quite free; sometimes threatening immediate suffocation. These remissions and exacerbations, Dr. Hastings remarks, continue throughout the disease, the state of the circulation varying with, and apparently depending on, that of the respiration.

"The disease is not stationary, the symptoms soon become more distressing. The aggravations of the difficulty of breathing are more alarming, and the remissions less perfect. The child often falls into a comatose state. A slightly livid tinge makes its appearance on the lips, from which the pallid cheeks are not entirely free. Even at this late period gleams of hope sometimes burst upon us. For a short time the difficulty of breathing may seem to subside, and the child to be better. But these hopes are never realized; even the next exacerbation of the symptoms may terminate in suffocation." 170.

In urgent cases the disease runs its course in about 72 hours; in the more protracted instances it usually terminates in less than five or six days.

§ V. In this section our intelligent and observant author traces the connexion of bronchitis with cutaneous diseases; but this subject was discussed at the commencement of this paper, and need not be entered upon here.

§ VI. In the sixth section Dr. Hastings takes up complications of bronchitis with diseases of the abdominal viscera. This combination, he observes, is frequently seen in the second variety (§ II.) of bronchitis, arising, as it often does, in habits deteriorated by intemperance.

"In these cases," says Dr. H. "a disease of the liver is brought on, which, by the irritation it produces in the mucous membrane of the lungs, often excites inflammation there. If this occur, we have, in addition to the pulmonic symptoms above described, the various symptoms indicating a languid hepatic affection." 177.

Here there is fulness with tenderness in the right hypochondrium, sense of oppression at the stomach, nausea, bitter taste in the mouth, giddiness or pain in the head, the fæces being generally dark-coloured and fetid. It is no uncommon occurrence for the more severe bronchial inflammations to be accompanied with symptoms of acute hepatitis, especially where the pulmonic affection has succeeded to measles. Gastric inflammation is occasionally combined with bronchitis, and then we have debility, pain in the stomach, increased by ingesta or pressure, together with the usual symptoms of gastritis. The same may be said where peritonitis occurs in combination with bronchial inflammation.

But Dr. Hastings thinks that inflammation of the larynx and upper part of the trachea is the disease most frequently found in conjunction with bronchitis.

"The symptoms," says Dr. Hastings, "which denote the first attack of chronic inflammation of the trachea, which is very insidious in its approach, are a tickling cough and tenderness in some part of the trachea, pressure generally producing a cough. The voice is always changed, the patient often speaking in hoarse whispers. The pulse is accelerated, fever for the most part attends, and a dense mucus is often expectorated.

"If the disease proceed to ulceration, the voice becomes more affected, the pulse is greatly accelerated, and the cough is harrassing, and attended with a copious purulent expectoration. The countenance expresses great anxiety, and the respiration is laborious; but no pain is felt in any part of the chest, though it is often referred to the epigastrium, when the dyspnœa has continued long." 179.

During this chronic disease, exposure to cold or other cause, may light up acute inflammation, which spreads along the whole of the mucous membrane, and then we have the symptoms of bronchitis mingled with those of chronic tracheal disease. We have then, superadded to the symptoms described above, a distressing straitness across the chest, a more aggravated dyspnœa, sometimes a slight lividity of the lips, and a more severe fever. If, by vigorous measures, the inflammation be checked, the original chronic affection of the trachea either goes on slowly to fatal disorganization, or yields, though seldom, to a long course of medicine.

Tumours, as bronchocele, occasionally by pressure on the windpipe involve the whole mucous membrane of the lungs in inflammation. It must also be candidly confessed that the features of bronchitis, especially when the disease is combined with other affections, are many times so faintly or obscurely marked, that it is with difficulty, or not at all, we are able to recognize them.

"Sometimes it is complicated," says our intelligent author, "with a multitude of other phenomena. Sometimes its commencement is imperceptible, its progress insidious and obscure, and its termination slow. Sometimes scarcely any of the symptoms of bronchitis appear; the patient is visibly affected with some other disease, which seems to prove fatal; and the inflammation of the bronchia is not discovered till after death. Sometimes its abrupt attack, its rapid course, and its combination with inflammation of the lungs, pleura, pericardium, or heart do not allow us, with any degree of certainty, to fix the character or seat of the affection." 182.

*Appearances on dissection.* These, of course, are various, according to the different forms of the disease. In the more acute cases, the lungs do not collapse on opening the thorax, although the parenchymatous structure be sound. On slitting open the trachea "it is often found full of fluid, (says Dr. H.) which is sometimes purulent, and at others consists of serous matter, with which coagulable lymph or mucus is generally mixed. The bronchia are, for the most part, plugged up by purulent matter, or tenacious mucus, or bloody serum." 182. When the substance of the lung is cut into, a frothy fluid escapes. In a few instances, Dr. H. observes, no fluid was found in the air passages, but only inflammation of the mucous membrane, which is always present. The capillary vessels of this tissue are invariably red and dilated; sometimes so much so, that this membrane "appears like a congeries of blood vessels." When the disease is less rapid in its progress, the whole of the mucous membrane is not always inflamed. Patches of inflammation are surrounded by portions of sound membrane, while a considerable quantity of serous fluid occupies the air cells. This is particularly the case in those who die of rubeola; but, "when bronchitis (says our author) succeeds to pustular cutaneous diseases, the mucous membrane is sometimes affected with minute ulcerations, and the whole of its texture appears much redder than natural." It is not to be supposed that even in acute bronchitis the inflammation is always bounded by the mucous membrane. The structure of the lungs is sometimes affected. They are either reddened, hardened, suppurated, or tuberculated. This is still more the case in chronic bronchitis, as we shall see farther on. The pleura too is not unfrequently inflamed, exhibiting, *post mortem*, a whitish incrustation, or an effusion into the cavities of the chest. The heart and its envelopes occasionally participate in the inflammation. "The membrane lining its cavities is more particularly the seat of inflammatory action. Coagulable lymph is occasionally found on the auriculo-ventricular valves. The cavities of the right side of the heart are, for the most part, larger, and contain more blood than usual." *Hastings*, *p.* 186. The stomach is sometimes found inflamed, and even ulcerated;—the liver in some cases indurated—of a lighter colour than natural, and enlarged, or its peritonæal coat inflamed and thickened.

"Every part of the peritonæum is now and then in a morbid state. Its whole surface is granulated; the intestines are united sogether and covered with a white incrustation; and a milky puriform fluid is contained in the cavity of the abdomen." 186.

In a section on the *nature* of the foregoing affections, Dr.

Hastings makes many reflections and observations which evince the soundness of his judgment—the best attribute of a physician. Dr. H. dissents from Dr. Badham, (whose work, by the bye, is a very valuable one, though too much overlooked by the profession at present) respecting the cause of the *wheezing* in bronchitis. Dr. B. attributed it to a certain constricted state of the inflamed parts, rather than to a redundant secretion of mucus. Dr. H. takes the contrary side of the question; attributing not only the wheezing, but the occasional exacerbations of dyspnœa, to the mechanical interruption which respiration experiences from redundant secretions in the air passages. Were we permitted to offer an opinion on the subject, we would say that the temporary exacerbations of dyspnœa were partly owing to the exuberant secretions, and partly to the difficult transmission of blood through the lungs when either their substance, coverings, or linings were inflamed. The dyspnœa, in what is termed spasmodic asthma, occurs long before the increased secretion of mucus, and, as Dr. Bree, in his excellent treatise on the subject, well explains, is owing to *irritation* in the lungs, whatever may be the cause of that irritation.* Now we all know, that wherever there is *irritation* there will be an increased *afflux of blood;* and the respiratory muscles being called into unusual exertion is the index of Nature's sufferings, and the effort she employs to obviate them, as Dr. Bree has long ago and ably explained. The headache in the second variety of bronchial inflammation is doubtless, in a great measure, owing to the "congestion of the blood-vessels of the lungs," which "necessarily interferes with the due return of blood from the head." The extreme debility attendant on acute bronchitis is readily accounted for by the state of the air-cells in the lungs, preventing the decarbonisation of the blood, and thus occasioning the lividity of the lips, and laborious respiration preceding the fatal termination. This interruption in the pulmonary function on the blood must also affect the brain and nervous system, and thus the functions of the whole body are drawn into disorder.

*Treatment of Acute Bronchitis.* Broussais and Hastings exhibit the following concise view of the treatment in language so precisely similar that the English appears almost a literal translation of the French:—

Broussais. "Modérer l'effort du systême sanguin, s'il est outré,

---

* See the whole of Section II. in Dr. Bree's work, where great erudition and judgment are combined to elucidate the subject.

par la saignée générale et locale, par les boissons mucilagineuses et aqueuses, un peu acidulées, et par l'abstinence des alimens: favoriser doucement la transpiration et diriger les mouvemens vers l'extérieur par les topiques émolliens, dans la violence de l'éréthisme, par les rubéfians et les vésicans, lorsque la réaction vasculaire et l'activité du système nerveux diminuent, telles sont les indications générales qui s'offrent à remplir dans le début des inflammations sanguines de l'organe pulmonaire." 148.

HASTINGS. "To moderate the excitement of the sanguiferous system—general blood-letting, acidulated mucilaginous drinks, and abstinence from all stimulating food. To promote expectoration and perspiration—antimonial and saline medicines. To direct the fluids towards the surface, and relieve the congestion of the debilitated capillaries—local blood-letting, blisters, and rubefacients." 208.

Dr. Hastings observes, that although general blood-letting is by far the most powerful for diminishing the excitement of the system, yet it is not equally applicable to all the varieties of bronchitis. In the *first* variety, for instance (§ 1) it is seldom necessary to detract blood, unless the fever be considerable, when it may be done with safety. In the *second* variety, venesection is generally proper, though to be employed with caution, as Sydenham long ago observed.

"The abstraction of ten ounces of blood from the arm early in the disease, sometimes mitigates the symptoms, after which it is generally more safe to depend upon an attention to diet, proper expectorants, and local evacuations. The peculiar tendency to effusion often renders the treatment of this affection difficult, as we are sometimes deterred by this cause from pursuing the blood-letting when the inflammatory symptoms indicate its employment. In this event we must subdue the inflammation by those means which are least likely to bring on effusion." 209.

But as the *third* variety of bronchitis sometimes occurs in robust habits, occasioning violent symptoms, the short state of excitement quickly terminating in irremediable debility, so the small space of time allowed for antiphlogistic measures, must be seized, for the purpose of making a decided impression on the complaint. Here blood-letting should be boldly employed—from twenty to thirty ounces may be taken from the arm at the first venesection, unless symptoms of syncope supervene. Although few cases yield to one bleeding, yet the repetition must be determined by the symptoms of the disease, and by the strength of the patient.

When the disease attacks children, general blood-letting, as far as the strength will permit, should be employed. And we may here observe that children, from a year upwards, may almost always be bled from the arm, although practitioners have a very general idea, that at such early periods we cannot procure blood from the brachial veins. Dr. Hastings thinks there is an advantage as well as facility in opening the external jugular veins of children—"as the blood is taken from a vessel which pours its contents into the thorax." But surely the brachial veins transmit their blood to the same point, and almost as directly as the jugulars. If children do not bear bleeding as well as adults, still, when life is menaced, we have no alternative—without detraction of blood there is no hope, and even that powerful measure does not always arrest the progress of the disease. In combinations of bronchitis with chronic ulceration of the trachea, the debility is so great, that bleeding, to any extent, is rarely admissible.

Vomiting has been recommended by many authors.

> "The benefits," says Dr. Hastings, "which arise from emetics are two-fold: they unload the primæ viæ, thus removing causes of irritation; and they increase the expectoration, on which, as is above observed, the favourable issue of the case so much depends.
>
> "Again, when the disease occurs in young children, emetics appear to be extremely serviceable, the stomach and bowels being generally in a bad state, and the bronchia loaded with redundant secretion." 212.

Our intelligent author justly observes, that the aqueous solution of tartarized antimony is, in general, the best medicine for producing these effects, excepting in young children, where Ipecacuan is to be preferred. Antimony generally excites diaphoresis, and, in fact, opens most of the secretory vessels; thus reducing the whole mass of fluids, and consequently checking the inflammation of any structure. Dr. H. thinks they should be given in saline draughts, and that nitrate of potass should always be combined whenever there is much fever.

In the second variety of bronchitis some have recommended the more stimulating expectorants. They cannot, Dr. H. thinks, be proper where the heat is much increased.

> "In the advanced stage, however, when the inflammation is nearly subdued, and the bronchia are clogged, ipecacuanha combined with squills is often of great service; but in all cases of this disease, as long as the excitement is considerable, if antimonial remedies be excepted, no expectorants are so useful as mucilaginous mixtures." 213.

Cathartics have not been considered beneficial in thoracic inflammations, as they were supposed to check expectoration. Our own experience has long ago taught us that, in common pulmonic inflammation they are exceedingly useful—anterior to the complete establishment of expectoration—that is, during the first days of the disease, as they not only powerfully deplete the whole system, but act by derivation from the pulmonary to the abdominal organs. When, however, Nature has fairly established expectoration, we hold it to be unsafe, because we have witnessed its bad effects, to act with any degree of force on the mucous surfaces of the bowels, since it very commonly interrupts the salutary discharge from the lining membrane of the lungs. If bleeding and purging are properly managed in the early stage of thoracic inflammation, there will rarely be profuse expectoration, and sometimes none at all. Dr. H. thinks there is less danger from purgatives in pectoral diseases than was once imagined, and directs that, in every variety of bronchitis, the bowels be kept lax, while at the commencement an active purgative should be administered to clear well the alimentary canal.

In the combination of bronchitis with abdominal disease, it is evident that the free employment of cathartics is of the utmost importance; particularly where the liver is affected, and its secretions deranged. Here we must always have recourse to cooling and mercurial purgatives. "From small doses of the neutral salts, given so as to keep up a constant catharsis, the greatest benefits ensue." *Hastings.*

"Mercurials, exhibited so as to act upon the system, are not usually beneficial; nevertheless, when in the sixth variety the liver is evidently affected, small doses of blue pill may be given, so that the gums may become tender." 214.

When bronchitis is combined with inflammation of the trachea, (producing symptoms resembling croup) Dr. Hastings thinks that analogy would lead us to employ calomel in frequently repeated doses. In one case (14th) where bronchitis occurred in conjunction with *chronic* inflammation of the mucous membrane of the trachea, great advantage arose from calomel and antimonial powder.

"The voice had become very indistinct, and the quantity of sputa was considerable. The patient took eight grains of calomel every day for several weeks, and eventually recovered."* 215.

In bronchial inflammation, as long as there is much fever, Dr. Hastings thinks opium is prejudicial. "But when that declines, and irritability of the system and air-passages still prevails, it not unfrequently allays the cough, and calms the

---

* In laryngeal inflammation of the *chronic* kind, we consider mercury a valuable auxiliary to the regular depletory measures, and counter-irritants. We may refer for a good instance to Dr. Hall's case of Laryngitis requiring laryngotomy, in the Medico-Chirurgical Transactions; or the second vol. of our *Quarterly Series*, p. 610.

patient. But opiates must be employed with great caution, especially in the *second* variety; for when the secretion is copious, and the strength much reduced, they interrupt, for a time, the efforts to expectorate, and may thus prove fatal."

"In combination with small doses of calomel, opium may sometimes be exhibited at an earlier period of the disease. When conjoined, these remedies not only diminish the cough, and assist expectoration, but seem likewise to regulate the secretions throughout the system." 215.

In the *second* variety, especially when a disposition to effusion is manifested, mild diuretics are serviceable But in all varieties of acute bronchitis, as Dr. Hastings justly observes, expectoration occasionally stops, apparently from want of power in the system; in which deplorable cases we must endeavour to support the strength and relieve the bronchia of the secretions with which they are clogged. Ammonia is a medicine the best calculated to effect the latter purpose, and in truth we have seen patients, as it were, snatched from impending suffocation by this valuable medicine.

Dr. Hastings appears to have entirely overlooked the effects of a regulated temperature of the patient's atmosphere, while labouring under acute bronchitis. This we consider to be a defect in a systematic work, and Dr. Hastings will doubtless allude to it in a future edition, many of which will ultimately be called for. Broussais particularly alludes to the necessity of a regulated temperature in the sick man's chamber:—"par un appartement convenablement échauffé on donnera à l'atmosphère ambiante une température qui favorise les evacuations cutanées.* The same able physician directs our attention to warm clothing, and especially flannel next the skin, to keep up a moderate degree of excitement there. The French physician having seen so many catarrhal inflammations protracted into a dangerous chronic state, apparently, in some cases, from debility, is more disposed to give tonics and mild nourishing diet, as soon as the expectoration becomes white and thick, than British practitioners are inclined to do.

"Aussitôt que l'expectoration blanche et épaisse annonce la résolution ou l'excrétion qui se décharge dans les bronches, on combine les toniques aux émolliens, on permet les alimens, et l'on ramène peu à peu le malade à son genre de vie accoutumé." 148.

In these lingering inflammations between the acute and chronic states, Broussais administered diluted red wines, and

* Tom 1. p. 149.

the decoction of cinchona lowered with mucilage—a medicine which he found preferable to all other tonics. Where intermittent fever is combined with bronchial inflammation—no rare case among soldiers in unhealthy situations—the combination is very difficult to manage; for if the fever continues, the bronchitis is aggravated by the cold fits determining the circulation to the interior organs, while bark or other tonics given to stop the ague, have generally the effect of heightening the local inflammation in the mucous membrane of the chest. In such circumstances, Broussais thinks it is more dangerous to exhibit stimulating febrifuges than to allow the intermittent to take its course for some time till the bronchitis is reduced.

But to come to the local means of cure. Topical blood-letting and blisters are of most importance in acute bronchitis. As general bleeding diminishes the excitement of the heart and larger vessels, so local detractions relieve the weakened and dilated capillaries—hence much advantage is derived from combining both measures. "Whenever therefore," says Dr. Hastings, "in bronchitis the symptoms require general blood-letting to be repeated, we should also have recourse to local evacuation." Blisters, our author observes, should not be applied "till the excitement has been considerably relieved by blood-letting." But when bronchitis occurs in phlegmatic habits, and assumes the form of peripneumonia notha, blistering may be employed from the beginning, and is one of the principal remedies to be relied on in that variety of the disease. In obstinate cases the blisters should be large, so as to cover the whole anterior part of the chest, and the surfaces kept open, or new ones applied.

The tepid bath, together with local fomentations or cataplasms, may also be used to remove the tension of the surface and excite diaphoresis. For the debility which almost always succeeds attacks of this disease, "nothing," says Dr. Hastings, "is more appropriate than a light nourishing diet with change of air." 218.

From page 218 to page 263 of Dr. Hastings's work, nineteen interesting cases of bronchitis are faithfully detailed, and very satisfactorily illustrate the principles and practice of which we have endeavoured to present an analysis to our readers. This part of the work we recommend to the *study*, not merely to the *perusal*, of our brethren, as containing a series of important and authentic documents which deserve their most serious attention.

---

Chronic Bronchitis. This disease, as Dr. Hastings truly remarks, embraces a wider field of investigation than we should have, at first sight, supposed. It is so often complicated with other affections, and so frequently gives origin to the most fatal disorganizations in the thoracic viscera, that it is entitled to a more rigid inquiry than it has hitherto obtained in this country at least.

Dr. Hastings divides chronic bronchitis, as he has done the acute, into several varieties—rather too many indeed, we think, for common practical purposes.

1. The first variety may be considered as the sequela of the acute form in old persons, attacking the patient generally at the commencement of cold weather, and lasting through the winter. The irritable mucous membrane is sensible to every atmospheric vicissitude—the respiration is uneasy, with occasional wheezing—the fits of coughing are most violent in the morning—the expectoration is copious, consisting of tenacious muco-purulent fluid, sometimes white and frothy. None, or only transient pains are felt in the chest—there are generally signs of disorder in the digestive organs, with sense of weight or pain in the epigastrium, white or loaded tongue, loss of appetite, quickened pulse, high-coloured or scanty urine, irregular bowels. Occasionally, however, chronic bronchitis exists for a long time without much constitutional ailment, or febrile action. This variety of the disease sometimes lays the foundation for hydrothorax, but more commonly subsides as summer advances.

2. The second variety resembles tubercular phthisis. The cough is severe, especially on lying down and getting up—the expectoration copious and part of it lumpy, varying in size from that of a pea to that of a bean, which lumps sink in water, and refuse to mingle therewith, being viscid, sometimes translucent, sometimes opake. With this expectoration there is often a flaky substance, not unlike the ramifications of the bronchia, which floats on the surface of the water. To these, Dr. Hastings remarks, there is usually added a third kind of matter, of a white, yellow, or greenish colour, which sinks in water, but by agitation may be broken into ragged portions, and somewhat diffused therewith. Lastly, some blood is often expectorated. The breathing is quickened, and often laborious, with a sense of oppression and tightness across the chest, although a full inspiration can be taken without pain. In bed in the morning the pulse seldom rises above 90; but when the patient sits up, the pulse is generally from 100 to 110, with an evening augmentation to 120. The temperature of the surface is pretty steadily above par, with a dryness and occasional roughness.

"In the night partial perspirations break out, which more especially appear about the head and breast. The desire for drink is always greater than in health. The urine is constantly high coloured, and deposits a copious red sediment. The face is often pallid, but there is sometimes a flush on the cheek, which more frequently appears during the evening febrile exacerbation. At the same time that the symptoms above enumerated occur, the patient visibly loses flesh and strength, and becomes more unequal to every kind of exertion." 268.

If the disease be not checked, a more alarming train of symptoms soon supervene. The debility and emaciation advance; the cough and expectoration increase—the latter sometimes to a pint or a pint and a half in the night—principally of a yellow or greenish colour, sinking in water, and diffusible, by agitation, therein. The dyspnœa is now distressing, as is the sense of tightness and weight across the chest, rendering the patient breathless on the slightest exertion. The pulse is now seldom under 120, and in the evenings it is more. "The tongue becomes cleaner, and, in many cases, all fur is removed: it assumes a shining appearance, and is redder than in health." There is nocturnal perspiration, leaving the patient much exhausted. "The body," says Dr. Hastings, "is inclined to constipation early in the disease; but, as the aggravated symptoms advance, a diarrhœa comes on, and, under these accumulated ills, the patient at length dies with all the appearance of tubercular phthisis." 270. Under proper management the second, or phthisical stage may often be prevented. In the advanced stage it not only resembles phthisis in appearance but in fatality.

The 3rd variety which our author describes, we shall pass over, as we really do not think it likely that it will often be distinguished in practice from the foregoing. Here, indeed, we conceive Dr. Hastings has refined with unnecessary minuteness—a thing that ought to be guarded against in all practical illustrations of disease. We shall also pass over the 4th variety of chronic bronchitis succeeding cutaneous eruptions; and the 5th, resulting from irritating substances acting on the mucous membrane, as in the cases of stone cutting and other unhealthy occupations, where hemoptysis is commonly produced; and where the disease disappears if the cause is removed, referring to the work itself for a more particular detail, in order that we may occupy more space with that variety of chronic bronchitis connected with diseases of the abdominal viscera.

6. One of the most common of these attendants of bronchitis is a chronic disease of the liver, of which our most intelligent author has introduced five interesting examples.

"It very often happens," says Dr. Hastings, "that in the commencement of this modification of the disorder we have no symptoms which can lead us to suspect any pulmonic affection. They are altogether such as are usually produced by hepatic disease. We have pain in the right hypochondrium, inability to lie on the left side, irregularity of the bowels, loaded tongue, and depression of spirits. The first warnings of disease in the bronchial membrane are very slight. There is a dry cough, unattended with any pain, which is often considered the necessary attendant on hepatic affection. By degrees the cough becomes more troublesome, and when it continues for some time, a tenacious mucus is expectorated. The breathing, too, is in some degree affected, and the patient complains of a weight and tightness across the chest. As the disease advances the cough is more troublesome, and the expectoration becomes copious: still, however, we do not trace much of the purulent character in the matter expectorated; it is principally mucus. The expectoration, however, in a more advanced stage, increases in an astonishing manner, and at length becomes purulent. Blood is not unfrequently mixed with the colourless matter, and sometimes pure blood is coughed up in the early stage of the disease; but in the cases of this kind which have been seen by the author, it is not a common occurrence." 277.

In these cases there is often a dull kind of pain, and always a tenderness in the epigastric region; and in progress of time, an irregular hectic is formed, differing, Dr. H. thinks, considerably from the true tubercular hectic.

"It is true there is usually some evening exacerbation, during which the face is generally flushed. The hands and face, also, are occasionally bedewed with perspiration in the night; but this, for the most part, goes off before morning. The emaciation becomes very perceptible, though it does not proceed so rapidly as in tubercular phthisis." 277.

With the above, the disorder of the digestive organs is well marked by flatulence, irregularity of bowels, furred tongue, impaired appetite, sense of fulness in epigastrio, "the dyspnœa and cough being always worse when the digestive organs are much oppressed." If the progress of the disorder be not checked, the symptoms approach still nearer to those of tubercular phthisis. Hectic fever becomes completely formed, and the patient is wasted with profuse perspirations, dropsical symptoms often preceding the fatal event.

Ulceration of the *stomach* occasionally is combined with chronic bronchitis; but here the emaciation is considerable *before* any marks of the pulmonic irritation are developed. Gastric symptoms also precede the thoracic affection. It is well known that symptoms resembling tubercular phthisis occasionally supervene on disease of the mesenteric glands.

In cases of this kind, Dr. Hastings thinks that accurate investigation will generally shew that the pulmonary affection is confined to the mucous membrane lining the bronchia and air-cells.

*Post Mortem Appearances.* Non-collapse of the lungs, on opening the thorax, with discharge of frothy fluid when an incision is made into their parenchyma. The capillaries of the mucous membrane are always dilated—"in the most marked examples the membrane is throughout like a congeries of vessels, and very much resembles the villous coat of the stomach or bowels, when a fine red injection has passed into its minute vessels" *Hastings*.

This injected state of the capillaries is sometimes only in patches, the intermediate spaces being nearly natural. The bronchial membrane is usually thickened, "and not unfrequently its surface is pulpy." It was not uncommon for Dr. Hastings to find this membrane ulcerated, especially where the irritation was caused by mechanical substances. The bronchia and air-cells are almost always filled with a purulent or muco-purulent fluid, mixed with bloody serum, and a frothy substance. When the disease extends beyond the mucous membrane, "the most common deviation from a natural state, (says Dr. Hastings) is a certain degree of thickening of the substance of the lungs, from which they become more solid." Tubercles are sometimes found in the lungs, when the mucous membrane is inflamed—they are sometimes in an incipient, sometimes a suppurated state. The pleura is often diseased. "Adhesions take place, and not unfrequently the whole of the serous membrane is full of tubercles." The heart is sometimes organically disordered, particularly in its valvular structure; and where abdominal disease have preceded the pulmonic attack, the liver is usually found in fault.

"It is generally enlarged and hard, and sometimes exhibits that peculiar nutmeg appearance which is so common. Its peritonæal coat is often very much thickened, and occasionally tuberculated. In some examples tubercles are also found in the substance of the liver; but this does not very frequently happen.

"When bronchial inflammation has succeded to long continued abdominal inflammation, the peritonæum is usually much diseased, being closely adherent to the bowels, and thickened. The whole of its reduplications on the viscera are sometimes thickly beset with tubercles of various sizes, some not exceeding a pin's head in magnitude, others as large as a pea. In some parts of the serous membrane, many of them being clustered together give it a very unnatural appearance for a considerable extent. The intestines are all closely united together, and their peritonæal covering is apparently much inflamed.*

"The cavity of the abdomen generally contains a quantity of yellowish fluid, which has flakes of gelatinous matter swimming in it.

"The mesenteric glands are frequently found much enlarged, and occasionally suppurated." 284.

Here ends the pathology of Dr. Hastings, with which intelligent author we have now, for some time, travelled in the strict analytical path, but from whom we must diverge for a moment to other sources of information on the subject under investigation.

Man, and the diseases by which he is assailed, are every where signally modified by the skies above him, the earth beneath him, and the host of moral and physical agents around him. In all cases, therefore, it is beneficial to take a comprehensive view of things, to prevent what Celsus complained of two thousands years ago:—"ex quo incidit, ut alia atque alia summi auctores quasi *sola* (auxilia) vindicaverint, prout cuique cesserant.†" In civil life, and in the bosom of peace, men are not exposed to such intense causes of disease, as in the tumults of war, and the enterprizes of emigration;—or at least if they are exposed to them, they have multiplied resources and means of counteraction. The pathology of diseases therefore varies, under different circumstances, and would appear to lead to different conclusions. Thus in the campaigns of the French armies, on the Continent, Broussais found that the almost universal pathological character of chronic bronchitis, or catarrhal inflammation, was an *induration of the parenchymatous structure of the lungs*, produced, he thinks, principally, by repeated exposures of the soldiers to cold and wet, under states of debility and privation.

"This chronic induration," he observes, "steals on in the intervals of great constitutional movements, although slight causes of irritation will readily kindle up the original storms. After seven, twelve, or fourteen days of pyrexia, the circulation becomes calm, the heat natural, the appetite returns, the complexion freshens, and strength appears to be fast returning. There only remains a cough, which gives little trouble during the day, but experiences an exacerbation at night. Frequently the cough is dry and husky; sometimes accompanied by much expectoration. In this state the patient goes about his affairs for a fortnight or a month; but now, on reflection,

---

* These appearances are similar to those so faithfully described by Dr. Baron in his work on "Tuberculated Accretions of Serous Membranes."

† Celsus, Lib. iii. cap. i.

he finds that he loses strength, instead of gaining it—that his breathing is very laborious on going up an ascent. His physician will perceive a slight acceleration of the pulse, and a flushing of the cheeks, in the evening—gradually the complexion becomes pale and sallow—the face becomes bloated—the legs edematous, and the strength fails. All these symptoms go on increasing, especially the cough, which renders the night most distressing. Still the patient never desponds: and as in approaching his final goal, he becomes incapable of taking either exercise or nourishment, he is nevertheless calm, and apparently free from sufferings. Finally, in two, three, or sometimes four months, he becomes anasarcous all at once, and dies suddenly."*

The organic alterations which M. Broussais found after death, in these cases, were—a reddening and induration of the parenchymatous substance of the lungs, so as to present the appearance and consistence of liver, in the centres of which indurated portions were sometimes observed certain softened and pasty points, as though the structure had putrefied. The mucous linings and pleural coverings were occasionally in a diseased state, with effusions or false membranes in the chest. This induration he considered to be always the effect of an irritation, of longer or shorter duration.

"Ce point d'irritation a pris naissance dans les capillaires de la muqueuse. Nons avons observé que, né dans les capillaires de glandes muqueuses, le point d'irritation ne se que trop souvent propagé, d'abord dans toute la membrane, d'où il a, par suite, envahi le parenchyme. Arrivé a son dernier periode, il a transformé tout le poumon en une masse rouge, composée, autant que les sens peuvent nous en convaincre, de vaisseaux de toute espèce remplis d'un sang coagulé.—*ib.*

But to return to Dr. Hastings, under the head "Ratio Symptomatum of Chronic Bronchitis," our able and ingenious author makes many judicious observations, which our limits will not permit us to quote. The section on "Diagnosis" is also interesting, and this we cannot entirely pass over, without culling some valuable matter. The great object of diagnosis is between bronchitis and tubercular phthisis. But it is to be borne in mind that not only are these two affections extremely difficult of distinction in advanced stages, but they are often combined.

"Early in the disease, the absence of pain during inspiration, the capability of resting on either side in bed, (when there is no abdominal disease,) the wheezing noise in respiration, the leaden

---

* Phlegmasies Chroniques, Tom I. p. 144.

colour of the lips, and the pallidity of the countenance, the appearance of the sputa, consisting almost entirely of mucus, occasionally streaked with blood, are symptoms sufficiently well marked to distinguish chronic inflammation of the bronchia from tubercular phthitis." 290.

When in advanced stadia of the disease the symptoms usually indicative of tubercular consumption become intermingled with those of chronic bronchitis, "the peculiar pallidity of countenance still continues, and there is in the majority of cases no pain in the chest; or, if it come on, it is diffused over the whole chest. The dyspnœa is not so great as in tubercular phthisis, and the patient can take in a much larger volume of air into the lungs." These *plus* and *minus* distinctions we have always considered extremely unsatisfactory.

"The quantity of matter expectorated in this disease is much greater than in tubercular or apostematous consumption, except at the time a vomica gives way, when large quantities are brought up; but this excessive discharge does not continue long in the latter disease, whereas in chronic bronchitis we often see a pint or a pint and half of matter expectorated during the night, and that for a long period together. We have not a short tickling cough in bronchitis; it is deep and sonorous. The paroxisms of hectic fever are much less regular in chronic bronchitis than in tubercular phthisis. The perspirations are partial, confined, perhaps, to the breast and upper extremities. The emaciation too is, generally speaking, less in inflammation of the mucous membrane than in tubercular phthisis, though we do meet with cases in which the emaciation is as great in the former as in the latter affection. When the pulmonic symptoms have arisen from a diseased liver, it is a strong presumption that the seat of the disease is in the bronchial membrane; for in almost all cases of this description the mucous membrane is the part affected. When this combination occurs, the spirits are always depressed; and in addition to the usual pulmonic symptoms, we have in this form of the disease a painful and distended epigastrium, unnatural stools, and disordered digestion. The mouth is dry in a morning, and the tongue loaded. The fits of coughing are constantly excited when the stomach is overloaded, and are apt to come on when the patient is lying on either side in bed." 292.

*Treatment.* Broussais lays down two main indications in the cure of chronic inflammation of the mucous membrane of the lungs:—1st. to diminish the general excitability, and keep the circulation quiet; 2d. to draw the excitement and the fluids towards other organs, and particularly towards the skin. These we believe to be the grand objects to be kept in view. Of the means employed in the treatment of this complaint, Dr. Hastings observes that—"some act by directly enfeebling the force of the circulation; some moderate excitement by re-establishing the secretions throughout the system; others act on parts in the neighbourhood of that which is diseased; and a few are *directly* applied to the inflamed membrane." Both Broussais and Hastings give an unfavourable picture of the effects of *inhalation* of medicated liquids in diseases of the mucous membrane of the lungs; and were it necessary to strengthen such authorities, we could bring forward strong proofs, from our own personal observation, of the non-efficiency of these medicated local applications.

"Le seul corps étranger," says Broussais, "qu'on fasse parvenir dans les vésicules bronchiques, c'est l'eau en vapeur; mais il faut qu'elle soit chaude, et par sa temperature elle fait plus de mal qu'elle ne procure de soulagement par sa qualité relâchante. Je n'ai jamais vu résulter un grand avantage des fumigations. Tous ces moyens ne font que boursouffler la membrane, et augmenter le sentiment de plenitude et de compression pectorale.*"

We thing that M. Broussais has gone too far in condemning so decidedly the vapour of warm water as injurious in inflammation of the mucous membrane of the lungs, since we have seen considerable advantage derived from this measure; but we fear that what he says respecting medicated fumigations will be found too true. The vapour of tar is the only kind of inhalation which Dr. Hastings has tried.

"Not one instance has occurred in the author's practice of even temporary relief in tubercular consumption by the inhalation of this vapour: indeed, it has appeared in some examples of this kind to cause a very tiresome irritation in the glottis, which has increased the cough. This has not been so much the case in chronic bronchitis; in this disease it seems to assist other remedies in restoring the mucous membrane to its healthy secretion; and in some very obstinate cases the inhalation alone has appeared to remove the diseased action in the mucous membrane of the lungs. In other instances the inflammation has been aggravated and rendered more acute by it. From the author's experience of this remedy, it appears that when the habit of body is irritable, and the inflammation at all active, the symptoms are increased by its use; but if the disease have been long in a chronic state, and the habit of body be not irritable, relief follows its application." 309.

The above, we believe, will be found a fair exposition of the effects of tar vapour—at least it corresponds with what we have seen ourselves; and we had opportunities of witnessing these effects a few years ago, when the hopes of relief drew crowds of pulmonary invalids to one of our principal

---

* Broussais, Tom. I. p. 151.

naval dock yards, for the purpose of inhaling the vapour of tar on a large scale there, under the direction of a gentleman who published on the subject. Many phthisical patients, indeed, as is usual when any new remedy is tried, declared themselves wonderfully benefited, for a time; but this benefit was, in most cases, either purely imaginary, or merely owing to the unusual evacuation of the lungs by the long-continued fits of coughing occasioned by the irritation of the tar fume. The tubercular disorganizations kept on, of course, their regular march.

In respect to blood-letting, Dr. Hastings justly observes, that although pulmonary inflammations, above all other maladies, are relieved by this powerful remedy, yet, "when the disease has existed long in the bronchial membrane, a considerable change takes place in its structure, and general debility always occurs, so that we cannot, in all cases, employ it with success." Even in those cases, Dr. Hastings thinks, where the chronic cough presents itself in persons not very much advanced in life, and whose constitutions are strong, with full pulse, inclined to hardness and frequency, large blood-lettings are improper, and "it is, perhaps, better to employ local than general blood-letting, for the former can be more frequently repeated, and the vessels of the lungs are thus unloaded at less expense to the system."

When catarrh has existed a long time in young persons, and produced symptoms resembling those of incipient phthisis, venesection will be improper. In such cases, recovery is slow, and is rather to be expected from the conjoined operation of several remedies than from the sudden operation of depletion. Repeated local detractions of blood are the safest means of reducing the superabundant excitement in the air-passages. Broussais, the most energetic of our French brethren, does not even mention local or general bleeding in chronic inflammation of the mucous membrane of the lungs. He merely cautions his countrymen against the employment of tonics and stimulants in this disease, which were probably in vogue when he wrote. In fact, the continental practice is here, as on too many other occasions, very inert.

After the force of the circulation has been weakened, but not till then, Dr. H. thinks blisters and rubefacients may be applied to the chest. "They generally relieve the cough, and by the irritation they occasion on the skin, seem to lessen the superficial inflammation going on in the mucous membrane. By their action, also, the purulent secretion from the internal surface of the lungs is sometimes converted into the natural mucus of the part." In those obstinate cases resembling tubercular phthisis, the more permanent drain and counterirritation of a seton or caustic issue, will be necessary. Broussais, and the continental physicians in general, attach much importance to topical applications to the chest. Broussais states that he had cured obstinate and long-continued catarrhal inflammations by large poultices to the front of the thorax. "Le soulagement fut si prompt, qu'il surpassa mon attente." "I have always," says he, "employed this remedy when circumstances permitted." "I prefer cataplasms to blisters when the patient appears, at the same time, nervous, irritable, and yet disposed to plethora." These cataplasms, he thinks, are superior to fomentations, as they are more manageable, and less apt to produce a chill, by wetting the patient's linen or bed-clothes. The same author highly praises rubefacients of lytta or mustard, in bronchitis. Issues are so important, that M. Broussais considers that physician reprehensible who has lost a patient without employing them. He observes, however, that, instead of being of service in *ulterior* stages, they hurry on the fatal event. It is at the point where acute inflammation is terminating in chronic, that issues are peculiarly serviceable. Caustic issues and setons, M. Broussais thinks, are nearly equal in point of utility. He tried cauteries in some cases with success; but does not speak very decidedly on their efficacy.

As in that variety of chronic bronchitis denominated "tussis senilis," the cough and dyspnœa are much aggravated by the redundant and accumulated secretions in the air-cells and passages, so emetics, by dislodging these secretions, are useful, as giving temporary relief. They have not, in Dr. Hastings's opinion, much effect "in forwarding a radical cure."

"The digitalis purpurea," says the same author, "does not seem applicable to all cases of chronic bronchial inflammation; but when it assumes the form of catarrhal phthisis, the fox-glove, by diminishing the velocity of the pulse, and by promoting a free discharge of urine, often relieves the breast. It is highly beneficial in all those examples in which bronchial inflammation has a tendency to terminate in dropsy, its powerful diuretic effects preventing the accumulation of the exhaled fluids; but when the disease is attended with much prostration of strength, digitalis, even if indicated, must be exhibited with great caution." 303.

Squill "is often serviceable when bronchitis assumes the character of chronic cough." Dr. H. has generally exhibited the powder in conjunction with ammoniacum, as in the pil. scill. comp. of the college pharmacopœia, and in old people, without much fever, found it of benefit. "The tincture of meadow saffron appears a very promising remedy in chronic bronchitis, and certainly possesses very remarkable powers.

It allays the cough, promotes the flow of urine, and keeps up a regular alvine discharge." Dr. H. thinks it can be given much more generally than squills, being less irritating, and therefore administrable where there is considerable fever, which, indeed, is relieved by this medicine, as it opens the secretions. Dr. H. usually gives twenty drops thrice a day, unless diarrhœa supervene, when the dose is diminished. If twenty drops produce no effect, it may be increased till the bowels, skin, or kidneys are acted upon. "If there be much quickness of the pulse, the author generally combines eight or ten drops of the tincture of digitalis with that of the meadow saffron; from which combination the cough is often relieved, and the quickness of the pulse is diminished."

Drs. Duncan and Armstrong have lately recommended certain vegetable balsams in chronic inflammation of the mucous membrane of the lungs.

"The latter thinks that the copaiba deserves to be conspicuously placed amongst the internal medicines, as it seems to exert a specific influence on the mucous membrane. It has failed with the author in producing so much benefit as he was led to hope from Dr. Armstrong's report. Whenever there is much fever, it appears to be increased by this remedy, and it does not always allay the cough, or alter the expectoration. It frequently disagrees with the stomach when given in sufficient doses to benefit the pectoral symptoms, and sometimes a diarrhœa comes on under its use. Occasionally it produces all these troublesome effects without relieving the cough. But this balsam certainly seems to exert an influence on the mucous membrane, although, perhaps, not a much greater one than the squills or meadow saffron. Further inquiry, however, may determine in what respects it is superior and in what inferior to these remedies. In the mean time Dr. Armstrong has done considerable service by bringing the copaiba into general notice." 305.

As the balsam of copaiba is very frequently adulterated, the want of success in Dr. Hastings's hands might have been partly owing to such circumstance.

A pill composed of extractum conii four grains, and pulveris ipecacuanhæ one grain, taken three times a day, often allays the cough, and produces a more healthy expectoration.

"There are some states of the system during the progress of chronic bronchitis in which cinchona may be useful; chiefly in those instances that succeed to acute bronchitis, where the debility brought on by the acute attack is very considerable. In such examples, if the dyspnœa be not increased, the benefits arising from its exhibition are sometimes very apparent. The profuse perspirations and other discharges are not only restrained by this remedy, but it occasionally appears to alter the secretion from the mucous membrane of the lungs, and thus brings about a more healthy condition of that membrane, by invigorating its blood vessels and restoring their natural tone. In these cases it may be combined with diluted sulphuric acid, which also tends to restrain the colliquative sweats that so often accompany this disease." 306.

Broussais observes that the cinchona sometimes proved useful; but he asserts that it is dangerous to continue it for any length of time—"parce qu'il n'est point ici le remede specifique." Pectoral ptisans, indeed, and diluent acidulated drinks with plenty of gum, appear to be the principal internal means which M. Broussais makes use of in this disease.

When chronic inflammation of the bronchia is combined with hepatic affection, Dr. Hastings recommends that "very small doses of calomel, or the blue pill, should be conjoined with the other remedies, and continued till the gums become slightly red, or till the appearance of the dejection is more natural, and the tenderness in the epigastric region is diminished." For the purpose of lessening the quantity of mercury in these cases, Dr. Wilson Philip has strongly recommended dandelion to be combined with it. Dr. Hastings remarks that this last medicine must be given in large doses, when, if the stomach bear it well, the flow of bile is promoted, and a healthy action of the bowels is kept up.

"If chronic bronchitis be combined with slow inflammation of the peritonæum and mesenteric glands, mercury may be carefully administered; but the disease generally resists this as well as every other remedy." 307.

The great utility of calomel in croup would tend to the hope of its being beneficial in chronic bronchitis; but the debility attendant on the latter disease is a strong objection. It is therefore chiefly used where the complaint is combined with abdominal affection.

"There are cases, however, in which it is advantageous in chronic bronchitis uncombined with any disease in the abdomen. These sometimes occur after measles, when the shrillness of the voice indicates considerable affection of the mucous membrane lining the trachea." 307.

Dr. Hastings and M. Broussais give different evidence respecting opium in chronic inflammation of the bronchial membrane. Dr. H. states that this medicine "can only be regarded as a palliative, and from its stimulating effects is often prejudicial, as long as there is much fever." In the chronic catarrh of old people, however, its use is sometimes, he thinks, unavoidable. "In such cases a full dose may be given at bed time, which will procure some rest in the early part of the night, before there is much accumulation in the bronchia and air-cells; but we must be very cautious in its use, as by preventing expectoration it sometimes proves fatal." *Hastings.*

"Opium," says M. Broussais, "is a precious sedative, when the gastric susceptibility (which we scarcely comprehend the meaning of in this case) will permit its use. If it be combined with ipecacuan powder, it promotes the cutaneous discharge, and suspends the cough for whole nights—an unspeakable advantage in chronic catarrhal inflammation. It is far better to prevent the suberabundance of bronchial secretion by opiates and mucilages combined with light tonics, than to be always endeavouring to expectorate it by squills and other acrid medicines, which only injure the already too sensible mucous membrane of the stomach. *Tom.* I. *p.* 154.

We have always looked upon the operation of opium in this class of complaints as rather preventive of *secretion* than of expectoration; and consequently after the febrile stricture is taken off, as unaccompanied with that danger which physicians so often speak of. Dr. Hastings himself remarks that "in the latter stages of chronic bronchitis, where the quantity of matter expectorated is very large, and the cough very troublesome, there is no remedy so powerful in allaying the uncomfortable irritation about the glottis as opium." He thinks it, however, not always free from inconvenience or danger, even in such cases.

Our readers are aware that Dr. Duncan, of Edinburgh, strongly recommends lactucarium as a substitute for opium, in many cases of pulmonary affection.

"Of all the medicines," says this venerable physician, "which I have employed for alleviating cough in phthisis, and indeed as a sedative in many other diseases, next to opium, I have found no article so beneficial as that substance which some have lately denominated lettuce opium, and which I termed lactucarium."* 308.

In the sixth volume of the college transactiens lately published, Dr. Louis Kerckhoffs states, that he administered, with great success, the white willow bark in powder, with sulphur in form of an electuary, to two patients who were apparently in the last stage of consumption, from affection of the mucous membrane of the lungs. Claret and nourishing soups were given at the same time.

For the effects of prussic acid in affections of the mucous membranes, we must refer to our analysis of two publications on this subject, in the present number of the journal.

---

* Observations on Pulmonary Consumption. By A. Duncan, senior, M.D. 2d edition, p. 162.

In the class of diseases under consideration, an attention to what has been quaintly termed the non-naturals is of more consequence than medicine. The diet must always be bland and unirritating, though it is not necessary to adopt the pure and refrigerating regimen necessary in acute diseases. In the early stages where there is much disposition to general vascular action, milk and vegetables form the proper aliment—in the more advanced stadia, when considerable debility has supervened, a more nutritious diet may be allowed. Cordial stimulating drinks are, of course, always improper.

The air which the patient breathes is of equal consequence with the food. The maxim of Celsus is peculiarly applicable to chronic bronchitis—"pessimum ægro cœlum est, quod ægrum fecit." Change of air often removes the disease when all other measures have failed. It is evident that as vicissitudes of temperature form one of the most frequent causes of the complaint, and are always apt to renew it, a warm and steady climate is extremely desirable. In this country a residence on the southern coast—especially about Penzance, for a few months in the summer, is to be recommended, as it sometimes invigorates the constitution, and restores the tone of the vessels on the bronchial surface, so as to prevent a return of the disease.

"In this climate," says Dr. H. "flannel next the skin during the spring and winter months, by slightly stimulating its vessels, sustains the circulation on the surface, and thus tends to relieve chronic inflammatory diseases of the pulmonary system." 311.

The bowels, of course, are to be regulated, "but purging is not beneficial." We doubt much whether we have not had too great a theoretical dread of purging when the mucous membrane of the lungs is carrying on the process of superabundant secretion. In two or three instances we have lately seen the balance of irritation beneficially shifted from the pulmonary to the gastric membranes, by smarter purgatives than we were formerly in the habit of giving. We throw out this hint for others to act on or not, as they may think proper.

Exercise, where it can be endured, is very serviceable in chronic bronchitis—especially horse and carriage exercise, as well as the swing, invented by Dr. Carmichael Smith.

We must pass over a great portion of Dr. Hastings's book, dedicated to the record of numerous cases illustrative of the principles which have been here laid down, in order to dedicate a page or two to the last chapter in the work, which treats of dropsy as dependent on inflammation of the mucous membrane of the lungs. Our readers will have perceived that Broussais makes dropsical infiltrations a very common attendant on the ulterior stages of bronchial inflammation—or rather of the condensations of lung which so often are induced by the disease. Dr. Hastings appears to take a proper view of this pathological phenomenon. He justly considers that the long continuance of the bronchial disorder must embarrass the function of the heart, not only by the mechanical obstruction which will be offered to the lesser circulation of the blood, but also by the non-purification of the blood itself in the lungs.* The appearances on dissection corroborate this supposition.

"The lungs and the right side of the heart are much loaded with blood. The bronchial membrane is always inflamed, its texture often thickened, and sometimes ulcerated. The bronchia and air-cells are usually filled with a frothy fluid, mixed with pus. Occasionally the lungs are otherwise diseased. There is generally a considerable quantity of fluid in the sacs of the pleura, and very frequently the pericardium contains more than six ounces of fluid.

"The heart is in almost every instance enlarged, and sometimes adheres to the pericardium. On examining its internal structure we often find some marks of disease. These in general are but slight, and arise from inflammation of its inner membrane, which produces some degree of thickening about the auriculo-ventricular valves. In some cases the disorganization is much more considerable, these valves being found almost cartilaginous." 382.

No sooner is the structure of the heart, particularly of its valves, diseased, than the venous system becomes congested, and dropsical effusions are the consequence. On the treatment of this variety of dropsy Dr. Hastings makes many judicious observations. He comes to the same conclusions with Dr. Crampton, respecting the indications afforded by the urine; namely, "that he has now forsaken it as a guide to the treatment of dropsy." He wisely observes that "we should rather be directed in the treatment by the state of the functions of the (internal) organs, and general inflammatory symptoms, than depend on the signs shewn by any one excretion." He does not venture quite so far as Dr. Crampton in blood-letting, though he evidently approves of the principle. He inclines more towards local abstractions of blood.

"Eight or twelve leeches applied to the chest sometimes do more in relieving congestions about the thorax than several ounces of blood taken from the arm; and the symptoms are thus relieved by a smaller expenditure of the general strength." 397.

Dr. Hastings does not wish, however, to underrate the effi-

---

* Till the dispute is settled respecting the oxygenation or decarbonization of the blood in the lungs, we might perhaps designate the process—*pulmonization* of the blood.

cacy of venesection. "He believes that in many examples of dropsy succeeding to bronchial inflammation, in which the heart is gorged with blood, and the venous system much congested, general blood-letting, and general blood-letting only, can save the life of the patient." Our author conceives that the indiscriminate use of drastic purgatives in dropsy has been productive of considerable mischief. In the variety of dropsical affection under consideration, these drastic purgatives appear to him inadmissible—excepting towards the close of the disease, when the infiltrations are fast advancing, and suffocation is impending. In such cases they are to be had recourse to for temporary relief. He trusts principally to purgatives of the saline class, given so as to produce a constant laxative effect. The supertartrate of potash is strongly recommended, especially when combined with the tartrate of potash and infusion of senna. In respect to diuretics, the following are our author's sentiments:—

"In this dropsical affection we frequently find that a copious flow of urine almost immediately succeeds the loss of blood, and the swellings occasionally disappear without any diuretic remedies having been employed. This, however, is not common; the more usual result is, that the increased flow of urine does not continue, if the venesection be not followed up by remedies which act on the kidneys. The author has principally used for this purpose the following diuretics,—squills, foxglove, meadow saffron, and crystals of tartar." 401.

With digitalis and squill he frequently combines small doses of the blue pill, especially where any hepatic disease is suspected.

"We are much indebted to those writers who have reprobated the indiscriminate use of mercury in dropsical diseases; but in this variety its administration, if kept within proper limits, is almost always beneficial. After the employment of blood-letting this mineral may not only tend to remove the congestion which invariably exists in the vessels of the liver; its general stimulating properties may also prove serviceable, by exciting the debilitated capillaries of the mucous membrane of the lungs to a more vigorous contraction." 405.

The meadow saffron, he thinks, deserves a fair trial in these cases. "In chronic inflammation of the bronchial membrane, it appears to regulate the secretion, to diminish the cough, and relieve the dyspnœa." It is evidently diuretic, and less stimulating than squills. The supertartrate of potash, however, should never be neglected; its mild saline diuretic and antiphlogistic qualities being peculiarly appropriate to the disease. Great care is necessary in the regulation of the diet, both during pulmonic affection, and in the convalescence which succeeds. After acute diseases the appetite is generally too keen, for some time, and blood is made too fast. Nothing is more common, in such cases, than relapses, from the weakened organ again taking on disease, especially where tonics are early and injudiciously given after the subsidence of the dropsical symptoms.

It is now high time to close this article, already rather too far extended. The numerous specimens of Dr. Hastings's work which we have laid before our readers, cannot but have impressed them with a high opinion of its own merits and its author's talents. It therefore needs no farther commendation from us, as the importance of the subject, and the ability with which it is handled by Dr. Hastings, will speedily induce the purchasers of select practical works to place the volume in their library, for reference as well as perusal. To our respected brethren on the Continent, especially M. Broussais, and to our able and zealous countrymen, Dr. Harrison and Mr. Alcock, we have been much indebted, in the construction of this eclectic article, and to them we offer the homage of our professional esteem.

